# Spatiotemporal variations of air pollutants based on ground observation and emission sources over 19 Chinese urban agglomerations during 2015–2019

**DOI:** 10.1038/s41598-022-08377-9

**Published:** 2022-03-11

**Authors:** Tianhui Tao, Yishao Shi, Katabarwa Murenzi Gilbert, Xinyi Liu

**Affiliations:** 1 College of Surveying and Geo-Informatics, Tongji University, Shanghai, 200092 China; 2 Zhejiang Zhipu Engineering Technology Limited Company, Huzhou, 313000 Zhejiang China

**Keywords:** Environmental social sciences, Urban ecology

## Abstract

The "comparative attitude" of urban agglomerations involves multidimensional perspectives such as infrastructure, ecological protection, and air pollution. Based on monitoring station data, comparative studies of multispatial, multitimescale and multiemission pollution sources of air quality on 19 urban agglomerations during the 13th Five-Year Plan period in China were explored by mathematical statistics. The comparison results are all visualized and show that clean air days gradually increased and occurred mainly in summer, especially in South and Southwest China. PM_2.5_, PM_10_ and O_3_ were still the main primary pollutants. PM_2.5_ is mainly concentrated in December, January and February, and PM_10_ is mainly concentrated in October–November and March–April. The O_3_ pollution in the Pearl River Delta and Beibu Gulf urban agglomerations located in the south is mainly concentrated from August to November, which is different from others from May to September. Second, from 2015 to 2019, the increasing rate of O_3_ concentration in any hour is higher than that of particulate matter (PM). Diurnal trends in O_3_ concentration in all directions also showed a single peak, with the largest increments that appeared between 13:00 and 16:00, while the spatial distribution of this peak was significantly regional, earlier in the east but later in the west. Third, this analysis indicated that the annual average air quality index (AQI) showed a gradually decreasing trend outward, taking the Central Plain urban agglomeration as the center. The ambient air pollutants are gradually moving southward and mainly concentrated in the Central Plains urban agglomeration from 2015 to 2019. Furthermore, in each urban agglomeration, the cumulative emission of PM_2.5_ is consisted of the four average emissions, which is approximately 2.5 times of that of PM_10,_ and industries are the main sources of PM_2.5_, PM_10_ and VOCs (volatile organic compounds). VOCs and NO_X_ increased in half of the urban agglomerations, which are the reasons for the increase in ozone pollution. The outcomes of this study will provide targeted insights on pollution prevention in urban agglomerations in the future.

## Introduction

In global ecological protection and urban development, air pollution has become a crucial issue affecting human health and the environment, along with climate change, which has attracted increasing attention from scholars and government administrators^1–4^. The number of air pollutants has grown from dozens to hundreds now. Major phenomena, such as acid rain, ozone holes, and urban heat islands, continuously challenge the airways of urban dwellers and sustainable urban development^5,6^. After decades of efforts by developed countries in Europe and America, sulfur pollution and soot pollution in urban air have basically been solved, and the environmental air quality has been greatly improved. However, the contribution of NO_x_ to environmental acidification is growing as the tropospheric ozone problem is aggravated by the emission of nitrogen oxides (NO_x_) and volatile organic compounds (VOCs)^7^. In recent decades, the enormous emissions of airborne particulate pollution have become the primary cause of climate change and air pollution in China^8^. Air pollution has gradually changed from a local problem to a regional problem and may be either emitted directly (primary pollutants) or formed in the atmosphere^9^. Major metropolises have invested large human and financial resources to control particulate pollution, but the effect is still unsatisfactory. CO, NO_x_, hydrocarbons and photochemical smog pollution remained serious.

Urban agglomeration (UA) is a crucial phenomenon at the urban scale and form in the process of globalization. In different stages of socioeconomic and human development, scholars use different terms to describe this urban landscape phenomenon, such as megalopolis^10^, urban clusters^11^, metropolitan chain areas^12^, urban agglomerations^13^, metropolitan interlocking regions^14^ and conurbations^15^. Despite the inconsistency in terminology, this extensive, multicentered, multicity urban landscape has been well recognized. Urban agglomerations (UAs) in China are dynamic regions with immense potential for regional development, which play a vital role in global competition and the international division of labor and gather many industries, transportation and residents, and the mechanisms of environmental pollution are more complicated^8,16^. Recently, in China, air pollution incidents have changed from frequent occurrence to visible improvement. Meanwhile, the characteristics and influencing factors of air quality have attracted the attention of many researchers^17–19^. Together these results indicate that the spatiotemporal patterns of air pollution are not only related to meteorological conditions such as terrain^20^, temperature^21^, and humidity^22^, but also to the emissions of local and surrounding cities, exhibiting distinct regional features ^23^. For example, source contributions to PM_2.5_ indicate that coal^24^, biomass burning^25^, transport^26^ and industry^27^ are the main sources.

Only three air pollutants, SO_2_, NO_2_ and PM_10_, were monitored, and the air pollution index (API) was calculated by the China National Environmental Monitoring Centre before 2013. Then, China promulgated the Ambient Air Quality Standard (GB3095-2012), adding three indicators, PM_2.5,_ CO, and O_3,_ for air quality evaluation. The air quality index (AQI) was used to evaluate the air quality, which makes the air quality evaluation more accurate and strict. At present, China's air quality evaluation indicators are mainly PM_2.5_, PM_10_, SO_2_, NO_2_, CO and ozone. However, in terms of identifying regions with similar air pollution behaviors and locating emission sources, few studies have comprehensively considered the spatiotemporal comparison of primary pollutants among UAs and detailed emission sources. For many countries, without this comprehensive assessment, it is impossible to understand the synergies and interrelationships between energy-related urban sprawl and air pollution. Previously published studies on spatiotemporal variations in PM_2.5_ and O_3_ have mainly focused on a specific region in China (Beijing-Tianjin-Hebei, Yangtze River Delta, or Pearl River Delta) and shorter observation times, ignoring UAs with weak economic development, such as northwest and central China^28–30^. Few studies have compared the changes in pollutant concentrations and primary pollutants in multiple UAs. Six air pollutants and 19 UAs were considered comprehensively in our work. Regional joint prevention and control of air pollution should be strengthened. As China has entered the 14th Five-Year Plan period, it is necessary to review and summarize the air quality situation of China's key UAs in the 13th Five-Year Plan (FYP) and study the impact of anthropogenic emission sources generated in urban construction on air pollution. This is a research highlight of this paper. Given these facts and based on air quality monitoring data, this study aims to investigate (1) the temporal variation characteristics of primary air pollutants in 19 UAs during China's 13th Five-Year Plan period, (2) the spatial distribution features of the barycenter of six ambient air pollutants from 2015 to 2019, (3) the dissimilarity of the contribution of atmospheric pollutants produced by anthropogenic sources at the UA scale.

This paper is organized as follows. In the first stage, we described the study area and the datasets used in the study. Then, we will present the spatial–temporal variation characteristics of primary pollutants based on UAs and anthropogenic source emissions. Finally, we summarized key research findings and drew conclusions.

## Study area and methods

### China’s urban agglomerations

**Figure 1:**
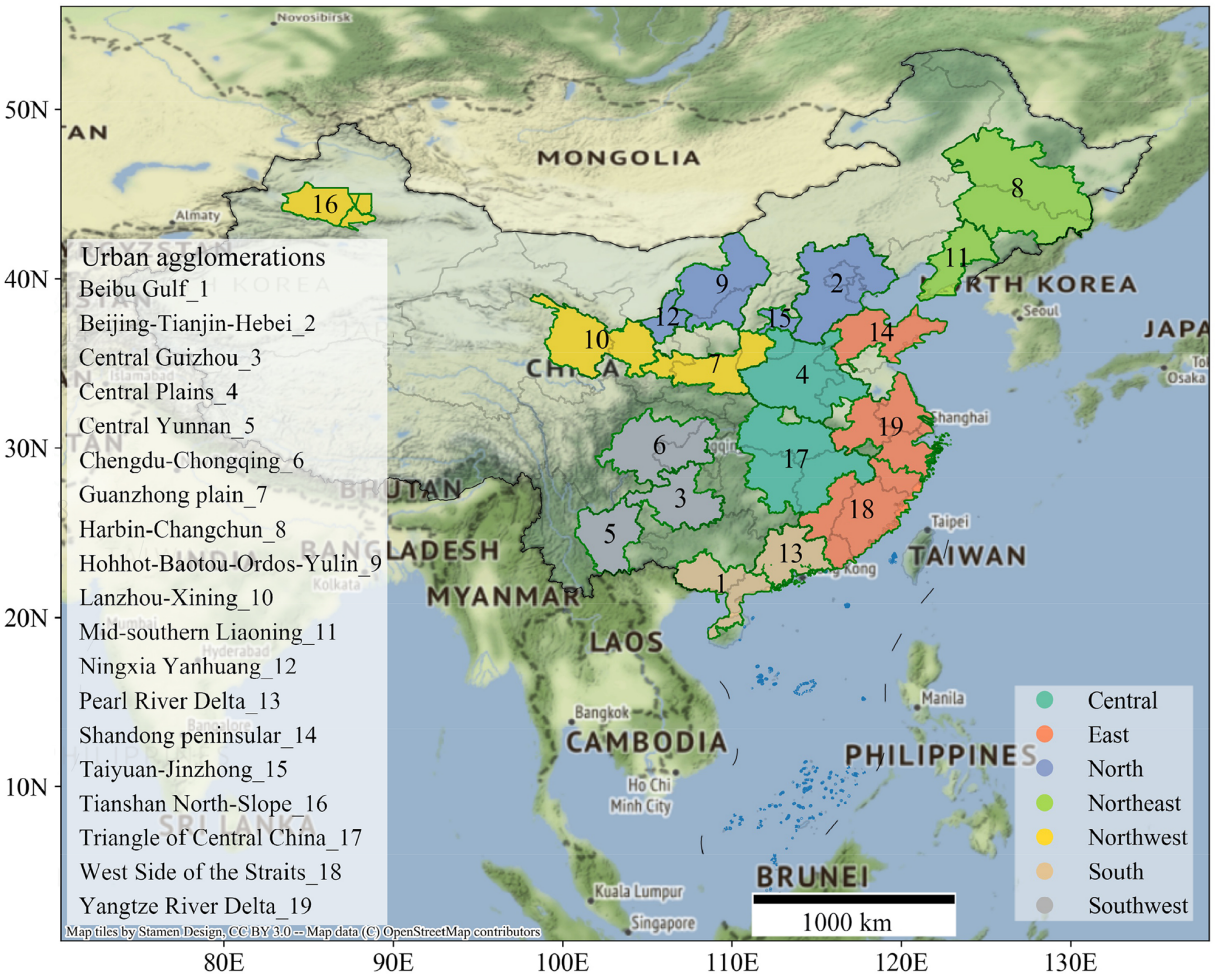
Geographical locations of 19 UAs in China. This figure was generated with python 3.7.6 based on geopandas and contextily packages. The scope of 19 UAs were obtained according to the Chinese planning documents. The source of the base map from contextily package is ‘http://{s}.tile.stamen.com/terrain/{z}/{x}/{y}.jpg’.

Due to the compound and regional features of air pollution, UAs are the main unit of joint prevention and control of air pollution. UA development planning in China can be divided into world-class, national and regional levels. The 19 selected Chinese UAs from south to north and from west to the east are Beibu Gulf, Pearl River Delta, Central Yunan, Central Guizhou, Chengdu-Chongqing, Triangle of Central China, Central Plains, West Side of the Straits, Yangtze River Delta, Shandong Peninsula, Tianshan North-Slope, Lanzhou-Xining, Guanzhong Plain, Ningxia Yanhuang, Hohhot-Baotou-Ordos-Yulin, Taiyuan-Jinzhong, Beijing-Tianjin-Hebei, Mid-southern Liaoning and Harbin-Changchun UAs. Nineteen UAs locations and names are annotated with colors and numbers in Fig. 1. Moreover, for convenience of expression, Table 1 briefly lists the basic indicators of the 19 UAs in China.

**Table 1:** Basic indicators of UAs in China.

Urban agglomeration (abbreviation)	Corecities	Level	Functions of UAs	Stations number	Key cities
Beijing-Tianjin-Hebei (BTH)^N^	Beijing, Tianjin	National	China's political and cultural center	79	13
Taiyuan-Jinzhong (TJ)^N^	Taiyuan	Local	The most dynamic economic belt in central Shanxi province	12	2
Hothot-Baotou-Ordos-Yulin (HBOY)^N^	Hothot, Ordos	Local	National high-end energy and chemical industry base	23	4
Ningxia Yanhuang (NY)^N^	Yinchuan	Local	Concentrating 90% of the urban population of Ningxia and spreading along the Yellow River	16	4
Shandong Peninsular (SP)^E^	Qingdao	Regional	The economic center of the Yellow River basin	89	14
Yangtze River Delta (YRD)^E^	Shanghai, Hangzhou	National	The leader of the Yangtze River Economic Belt	170	27
West Side of the Straits (WSS)^E^	Xiamen, Wenzhou	Regional	New comprehensive corridor for opening to the outside world along the southeast coast	88	19
Central Plain (CP)^C^	Zhengzhou	Regional	The most densely populated one with rapid industrialization and urbanization	110	28
Triangle of Central China (TCC)^C^	Wuhan	National	An essential part of the Yangtze River economic belt and a key area to promote new urbanization	138	31
Pearl River Delta (PRD)^S^	Guangzhou, Shenzhen	National	China Open and Innovation Pilot Zone	72	15
Beibu Gulf (BG)^S^	Zhanjiang	Regional	Chinese most growing coastal economic belt	41	15
Harbin-Changchun (HC)^NE^	Harbin, Changchun	Regional	Core competitiveness and essential influence in Northeast China	55	11
Mid-southern Liaoning (MSL)^NE^	Dalian, Shenyang	Regional	A region of earlier industrial development and a high level of urbanization	57	9
Central Guizhou (CG)^SW^	Guiyang	Local	Pilot demonstration area of new urbanization with mountainous characteristics	23	6
Central Yunnan (CY)^SW^	Kunming	Local	China's southwest economic growth pole	17	5
Chengdu-Chongqing (CC)^SW^	Chengdu, Chongqing	National	China's largest economy closest to Southeast and South Asia	88	16
Guanzhon gPlain (GP)^NW^	Xi’an	Regional	The second largest urban agglomeration in the western region	51	10
Lanzhou-Xining (LX)^NW^	Lanzhou, Xining	Local	A major area for national security and ecological security	19	9
Tianshan North-Slope (TNS)^NW^	Urumqi	Regional	Chinese westernmost urban agglomeration and the core area of the silk road economic belt	13	10

N, E, C, S, NE, SW, NW correspond to North, East, Central, South, Northeast, Southeast, Northwest.

### Data sources and statistical analyses

Real-time daily hourly concentrations in each monitoring site covering 19 UAs (Table 2) were derived from China's National Environmental Monitoring Centre. In addition, city daily data (excluding detailed sites) were also used for verification. In this study, the monitoring data were pretreated with python language programming based on Spider platform to ensure the effectiveness of air pollutant concentration data. First, some outliers, such as hourly pollutants concentrations in each station less than zero and missing values, were removed. Secondly, the daily average value of each pollutant in each station was only calculated when the valid data of the day in each station is greater than or equal to 20 h. And the number of effective monitoring points shall not be less than 75% of the total number of urban points. Then the daily mean value (i.e., arithmetic mean of a 24-h monitoring value on a natural day) of urban pollutants can be obtained by calculating the average of each pollutant at each station. Finally, month and annual mean pollutants concentrations for the monitoring sites and UAs were obtained according to the arithmetic mean method^31^. Comparing the annual average concentration of the city with the government report, our calculation results were found to be reliable.

**Table 2:** The raw data example of cities.

Time(y-m-d h)	City	Site	AQI	PM_2.5_ (μg/m^3^)	PM_10_ (μg/m^3^)	SO_2_ (μg/m^3^)	NO_2_ (μg/m^3^)	CO (mg/m^3^)	O_3__8h (μg/m^3^)
2015-01-02 01:00:00	Beijing	Olympic Sports Center	145	111	140	61	102	2.7	6
2015-01-02 02:00:00	Beijing	Olympic Sports Center	134	102	124	51	93	2.4	2
2015-01-02 03:00:00	Beijing	Olympic Sports Center	97	72	103	33	88	1.7	2
……	……	……	……	……	……	……	……	……	……
2015-01-02 01:00:00	Beijing	The temple of heaven	132	100	148	20	83	2.5	14
2015-01-02 02:00:00	Beijing	The temple of heaven	145	111	161	18	88	3	9
2015-01-02 03:00:00	Beijing	The temple of heaven	130	99	153	20	91	3.5	7
……	……	……	……	……	……	……	……	……	……

It is well known that 2020 is the year for China to fight theCOVID-19 pandemic with all their strengths^32^. Home isolation was adopted to reduce mobility, and social and economic development stagnated. Meanwhile, anthropogenic activities such as transportation and industry are directly proportional to air pollution. Therefore, the study period was conducted from January 1, 2015, to December 31, 2019, avoiding the impact of the COVID-19 epidemic. The air quality index (AQI) is a quantitative description of air quality data, which can denote the short-term air quality status and trends in a city^33^. The overall AQI represents the maximum of the sub-AQI of all pollutants, where when the AQI is higher than 50, the highest sub-AQI contributor is defined as the primary pollutant on that day^34^. The calculation of the AQI and primary pollutants is as follows:

1 $$ IAQI_{P} = \frac{{IAQI_{Hi} - IAQI_{Lo} }}{{BP_{Hi} - BP_{Lo} }} \times (C_{P} - BP_{Lo} ) + IAQI_{Lo} $$

2 $$ {\text{AQI}} = {\text{Max}}\left\{ {IAQI1, \, IAQI2, \ldots , \, IAQIp, \ldots ,IAQIn} \right\} $$

where IAQI_P_ is the air quality sub-index of pollutant item P. C_p_ is the concentration value of P. BP_HI_ and BP_LO_ are the high and low values of the pollutant concentration limit, respectively, similar to C_p_^35^. IAQI_HI_ and IAQI_LO_ are air quality sub-indexes corresponding to BP_HI_ and BP_LO_. After calculating the air quality sub-index of various pollutants, the maximum value is the air quality index (AQI). Primary pollutants are the largest corresponding pollutant items of IAQI_P_.

### Illustration of spatiotemporal variations in sector emissions

The emission source inventory used in the experiment is from the MEIC (Multi-resolution Emission Inventory for China) 2017 version developed by Tsinghua University, with a spatial resolution of 0.5° × 0.5° (http://meicmodel.org). MEIC is a bottom-up emission inventory model based on a cloud computing platform that covers ten air pollutants and carbon dioxide emissions (SO_2_, NO_x_, CO, NMVOC, NH_3_, PM_2.5_, PM_10_, BC, OC and CO_2_) . It should be noted that the emission inventory contains the location and the amount of emission sources information, but does not establish a direct relationship with ambient air quality. The emission source inventory here is mostly used as input data of various chemistry transport model^36^. Since pollutants go through a series of complex physical and chemical processes after entering the atmosphere, the contribution of various industries to the environmental concentration of pollutants is highly nonlinear and constantly changing. That is, emissions from MEIC are not equal to ground monitoring values.

The emission data are divided into five sectors: industry, power, residential, transportation and agriculture. Annual grid emission inventories of 0.25°, 0.5° and 1.0° spatial resolutions are provided. Currently, MEIC data are widely used by more than 100 research institutions and business units to forecast air quality and plan air pollution standards^36,37^.

Herein, in our research, two mathematical statistical methods, including the average industrial emission of pollutants (IEP) and growth rate, were used to evaluate spatiotemporal variations in IEP in 19 UAs. The formulas of these indexes are specified as follows:

3 $$ AI_{x,j} = \frac{1}{n}\sum\limits_{i}^{n} {IEP_{i,j} } $$

4 $$ GI_{x,j} = \frac{{AI_{x,j}^{2017} - AI_{x,j}^{2015} }}{{AI_{x,j}^{2015} }} \times 100\% $$

where *AI*_*x,j*_ is the average industrial emission of *j* pollutants in city *x*, *IEP*_*i,j*_ is the estimated *IEP* of pixel *i* of *j* pollutants, and *GI*_*x,j*_ stands for the growth rate of *IEP* of j pollutants. To further explore the spatial and temporal variations in UAs, the spatial changes in the industrial emissions of pollutants at the UA scale were calculated by averaging the *AI* value in the inner city. Specific experiments were completed by zonal statistics function on ArcGIS 10.6 platform. Zoning statistical tools are also widely used in scientific researches, which can summarize the values of raster within the zones of another dataset (either raster or vector). The explanation link for this tool is as follows: https://desktop.arcgis.com/zh-cn/arcmap/latest/tools/spatial-analyst-toolbox/how-zonal-statistics-works.htm.

Besides, Mann–Kendall trend test and spatial centroid calculation were used to explore the spatiotemporal variation patterns of UAs on pollutant concentration.

## Results and discussion

### Daily change in primary pollutants

**Figure 2:**
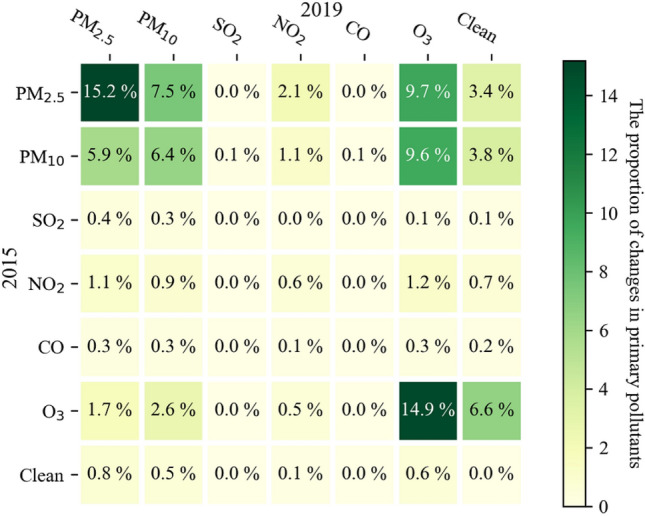
Transfer change matrix heatmap of primary pollutants from 2015 to 2019.

To elucidate the change trend of primary pollutants under the 13th Five-Year Plan, we calculated the daily primary pollutants in 2015 and 2019 based on formula (1) and formula (2). Such diurnal comparisons can reduce the effects of seasonal weather to some extent. From the 19 UAs (224 prefecture-level cities), the heat diagram of the daily change transfer matrix of primary pollutants from 2015 to 2019 is shown in Fig. 2, including six primary pollutants and clean day conditions.

From the sum of the diagonal numbers, 37% of the primary pollutants had no shift during the 13th Five-Year Plan period. PM_2.5_, PM_10_ and O_3_ were the main primary pollutants, especially PM_2.5_. More primary pollutants were diverted to ozone pollution, indicating that the proportion of O_3_ as the primary pollutant is gradually increasing. In addition, the proportion of clean air has increased significantly, which shows that pollution control has been effectively reflected during the 13th Five-Year Plan period. However, the proportion of NO_2_ before and after metastasis was approximately the same, with approximately 5% NO_2_ pollution. This may imply that the governance of NO_2_ pollution was rendered nonsignificant. It is noteworthy that ozone pollution in China has become an increasingly prominent task in recent years. Similar to Xiao’s^16^ research on ozone pollution, they argue that present-day ozone levels in major Chinese cities are comparable to or even higher than the 1980 levels in the United States. Taken together, ozone and PM_2.5_ have become the top two air pollution pollutants in China.

### Monthly distribution of primary pollutants

**Figure 3:**
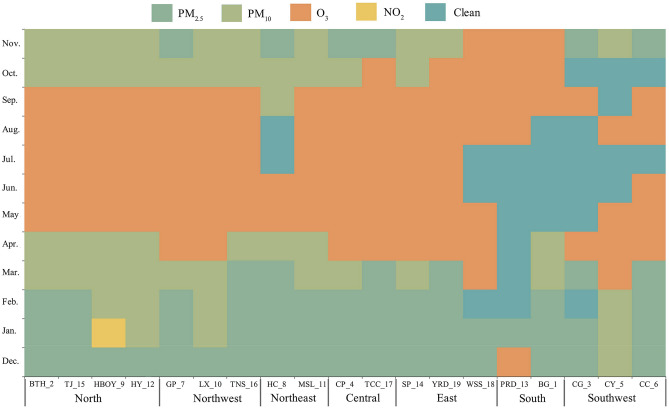
Monthly calendar of primary pollutants in UAs in 2019.

To further explore the spatiotemporal distribution of the primary pollutants across the UAs, we obtained the most primary pollutants per month by dividing the number of days with the most pollutants by the number of cities in each UA from the 2019 data. In Fig. 3, the UAs location was plotted on the abscissa, and the monthly variance of the primary pollutant was plotted on the ordinate. As shown in Fig. 3, PM_2.5_ appeared as dark green, PM_10_ appeared as light green, O_3_ appeared as orange, NO_2_ appeared as yellow, and clean days appear as dark blue. The main pollutants in the 19 UAs are PM_2.5_, PM_10_ and O_3_. NO_2,_ as the primary pollutant, only appeared in the HBOY UA in January. Ordos, located in HBOY, possess rich oil and coal resources, with coal mining as its leading industry^38^. According to the China Energy Statistical Yearbook 2019, nearly 250 million tons of raw coal were used for thermal power generation in Inner Mongolia Autonomous Region, making it the region with the largest amount of raw coal for thermal power generation in China^39^. To a certain extent, the increase of heating^40^ and the imperfect denitration technology^41^ are both contributing to the increase of NO_2_ pollution in the atmosphere. CO and SO_2_ did not become major pollutants. Clean days (where AQI < 50) occurred mainly in South and Southwest China during summer. As the primary pollutant, PM_2.5_ is mainly concentrated in December, January and February, that is, it occurs in winter. As the primary pollutant, PM_10_ is mainly concentrated in October–November and March–April, that is, autumn and spring. As the primary pollutant, O_3_ is mainly concentrated from May to September, that is, summer. These results are in accord with recent studies indicating that they showed a strong seasonality but there were small differences^6,42,43^.

From the perspective of UAs, monthly primary pollutants in different UAs are different^44^. The O_3_ pollution in the PRD and BG, as representatives of UAs in southern China, mainly concentrated from August to November. In addition to human factors, this may be associated with lower latitudes, higher temperatures and stronger solar radiation^19,45^. The northern, northwestern, northeastern, central and eastern UAs have similar monthly primary pollutant distributions, with PM_2.5_, PM_10_ and O_3_ pollution distributed in the 12 months of 2019. Near surface O_3_ is mainly produced by the complex photochemical reactions between nitrogen oxides, volatile organic compounds, carbon monoxide, methane and other precursors^19^. O_3_ was a major pollutant in most UAs from May to September, which may be the result of the comprehensive action of anthropogenic factors and meteorological conditions.

### Annual patterns of ambient air pollutants

**Figure 4:**
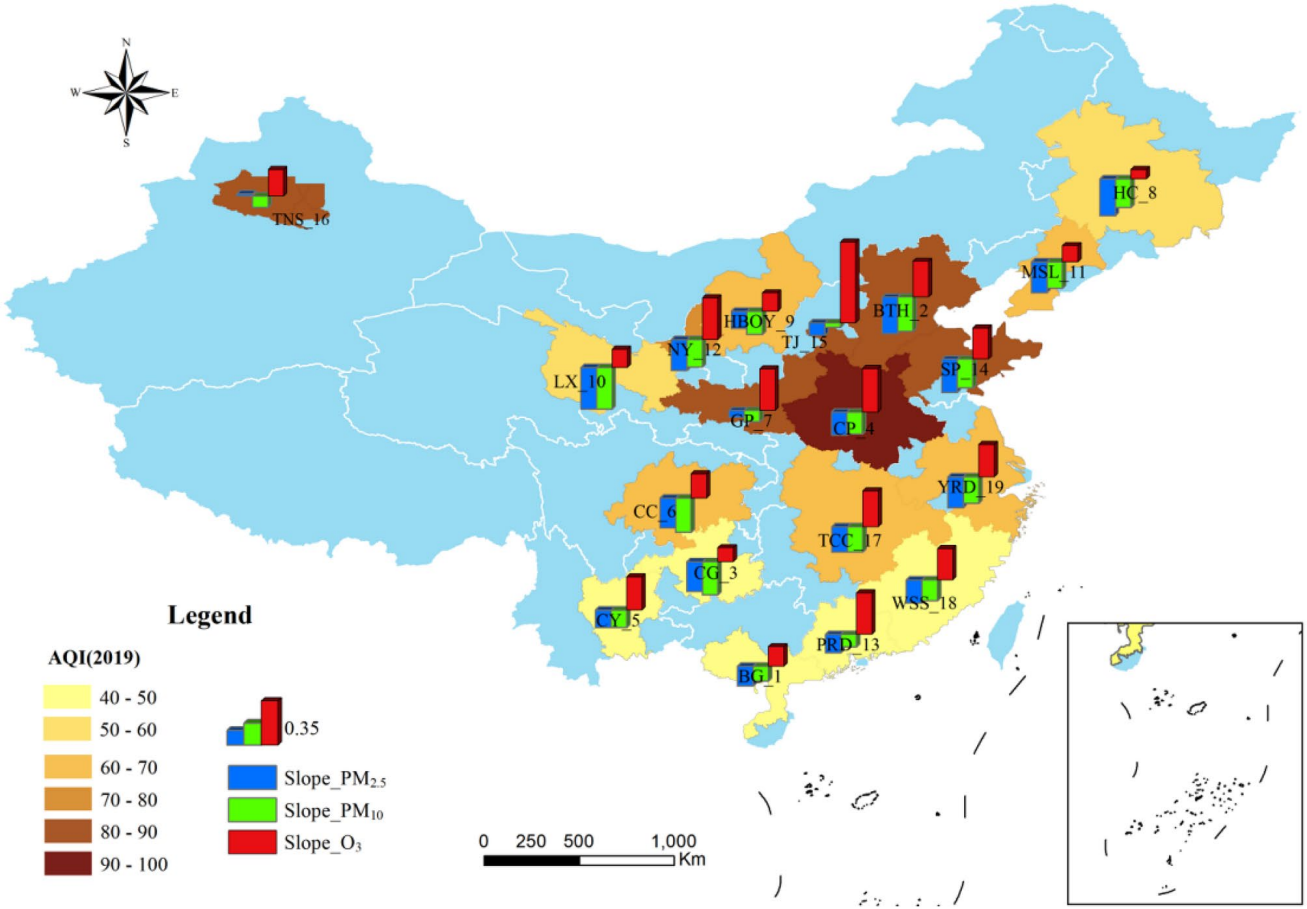
Annual variation trends (slope values) of three major pollutants in each UA of China from 2015 to 2019. This figure was made in the ArcGIS 10.6 platform.

As shown in Fig. 4, unary linear regression was used to extract the time variation trend of PM_2.5_, PM_10_ and O_3_ concentrations in each UA. The slope value depicted a decreasing trend in the annual mean PM_2.5_ and PM_10_ during 2015–2019, suggesting that the emissions of particulate matter were effectively controlled. Meanwhile, ozone pollution has become a new environmental challenge in most UAs. The non-parametric Mann–Kendall trend test and Sen’s slope estimator (MKTT-SSE) confirmed these findings (Table 3).

**Table 3:** MKTT-SSE for annual mean concentration of air pollutants in 19 UAs from 2015 to 2019.

UAs	PM_2.5_		PM_10_		NO_2_		O_3_		SO_2_		CO	
	MK (z,s)	SSE	MK (z,s)	SSE	MK (z,s)	SSE	MK (z,s)	SSE	MK (z,s)	SSE	MK (z,s)	SSE
BTH_2	−2*	−6.93	−2*	−10.07	−1	−2.97	2	3.04	−2	−6.78	−2*	−0.14
TJ_15	−1	−2.88	0	1.53	1	3.10	2	4.31	−2	−11.98	−2	−0.14
HBOY_9	−2	−1.21	0	−2.85	1	0.40	0	0.40	−2*	−2.64	−2	−0.08
NY_12	−2	−3.71	0	−3.46	1	1.12	1	2.30	−2	−8.32	−2*	−0.04
SP_14	−2	−7.99	−2*	−10.20	−2	−1.47	1	2.77	−2*	−7.93	−2*	−0.14
YRD_19	−2*	−3.50	−2	−4.65	−1	−0.64	1	1.34	−2*	−3.27	−2*	−0.05
WSS_18	−2*	−1.79	−2*	−1.97	−1	−0.71	1	1.89	−2*	−1.37	−2*	−0.04
CP_4	−2*	−4.50	−2*	−6.21	−1	−1.78	2	4.05	−2*	−7.04	−2	−0.17
TCC_17	−2	−3.16	−2*	−4.74	0	−0.26	2*	1.83	−2	−3.09	−2	−0.05
PRD_13	−1	−1.21	−1	−1.09	0	0.02	2*	1.72	−2*	−1.06	−2	−0.04
BG_1	−2	−1.17	−2*	−1.00	0	−0.05	1	0.62	−1	−0.45	−2*	−0.03
HC_8	−1	−3.95	−1	−5.09	−2*	−1.98	0	0.09	−2*	−3.65	−1	−0.05
MSL_11	−2	−4.02	−2	−5.62	−2*	−1.17	0	0.13	−2*	−4.94	−2*	−0.07
CG_3	−2	−2.22	−2	−3.36	−2	1.33	0	0.17	−2*	−1.52	−2	−0.04
CY_5	−2	−1.26	−1	−2.34	0	0.17	1	1.56	−2*	−2.43	−2	−0.05
CC_6	−2	−4.21	−2*	−7.67	1	0.12	0	0.93	−2*	−2.38	−2*	−0.05
GP_7	−1	−2.84	−1	−2.83	0	−0.14	1	2.27	−2	−5.89	−2	−0.17
LX_10	−2*	−4.14	−2	−6.96	−2	−0.62	0	−0.06	−2*	−2.48	−2*	−0.03
TNS_16	0	−0.27	0	1.74	0	0.17	0	1.05	−2	−1.36	−1	−0.13

*P < 0.05.

*MK* Mann–Kendall trend, *SSE* Sen's slope estimate.

The unit of SSE is concentration/year. Concentration: μg/m^3^ for PM_10_, PM_2.5_, O_3_, NO_2_ and SO_2_, and mg/m^3^ for CO.

In more details in UAs, the average annual PM_2.5_ and PM_10_ concentrations significantly decreased, mostly in the BTH, SP, PRD and CC UAs, which are economically developed regions in China^46,47^ (Fig. 4, Table 3). Among them, BTH region had the largest reduction of PM_2.5_ concentrations in the 10th, 12th and 13th FYP period^48^. In contrast, the annual mean ozone pollution displayed the greatest enhancement while PM_2.5_ and PM_10_ showed a minimal decrease in the TJ UA in northern China. Regarding MKTT-SSE, annual mean concentration of SO_2_ decreased statistically significant (P < 0.05) in most UAs, especially in TJ, which affiliated Shanxi Province, with nearly 12 μg/m^3^ per year during the 13th Five-Year Plan period. As reported by People's Government of Shanxi Province, the main measures to the reduction the concentration of SO_2_ include the designation of "no-coal areas", raising the standard for eliminating excess capacity and increasing railway freight^49^. However, the concentration of NO_2_ was still increasing with 3.10 μg/m^3^ per year, so it is imminent to adjust the industrial structure and control the discharge of pollutants. Regional emergency linkage can effectively deal with the heavily polluted weather. For CO pollution, it decreased statistically significant (P < 0.05) with the slope of 0.07 mg/m^3^ per year in all UAs (Table 4).

**Table 4:** Recommended AQG levels and interim targets.

Pollutants	Averaging time	GB3095-2012		AQG 2021				AQG
		Level-1	Level-2	IT-1	IT-2	IT-3	IT-4	
PM_2.5_ (µg/m^3^)	Annual	15	35	35	25	15	10	5
PM_10_ (µg/m^3^)	Annual	40	70	70	50	30	20	10

At the same time, by checking the annual average AQI in 2019, we showed that the CP UA has the highest annual average AQI, and the particulate matter pollution and ozone are both high, suggesting that the control effect is not obvious in the central region. We found that the annual average AQI showed a gradually decreasing trend outward, taking the CP UA as the center. In addition, the UAs in the southern and southwestern regions showed both good annual average AQI and low fine particulate pollution while high ozone concentration pollution. The average annual AQI, fine particulate matter and ozone pollution in the TNS are also at a high level in 19 UAs. This is consistent with the level of the monthly primary pollutants mentioned above.

**Figure 5:**
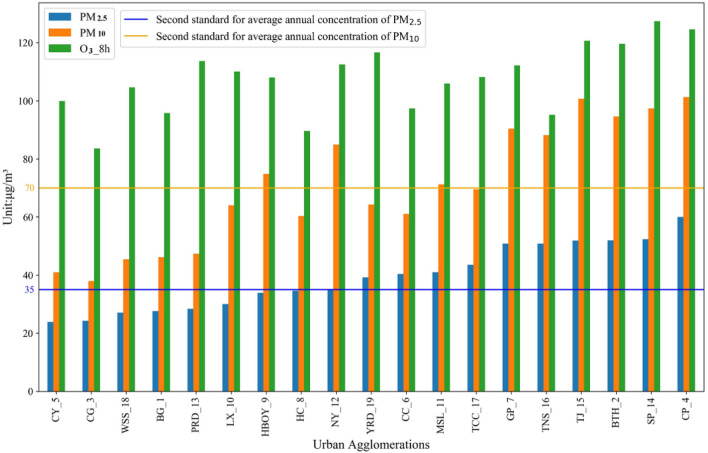
Numerical distribution of three major pollutant concentrations in UAs in 2019.

The World Health Organization (WHO) published new Global Air Quality Guidelines (AQGs), which recommend new air quality levels on 22 September 2021. As shown in Table 4and Fig. 5, almost half of the UAs have reached the second-level national standards of China and the first-stage interim target of the World Health Organization.

### Hourly variations in PM_2.5_, PM_10_ and O_3_

**Figure 6:**
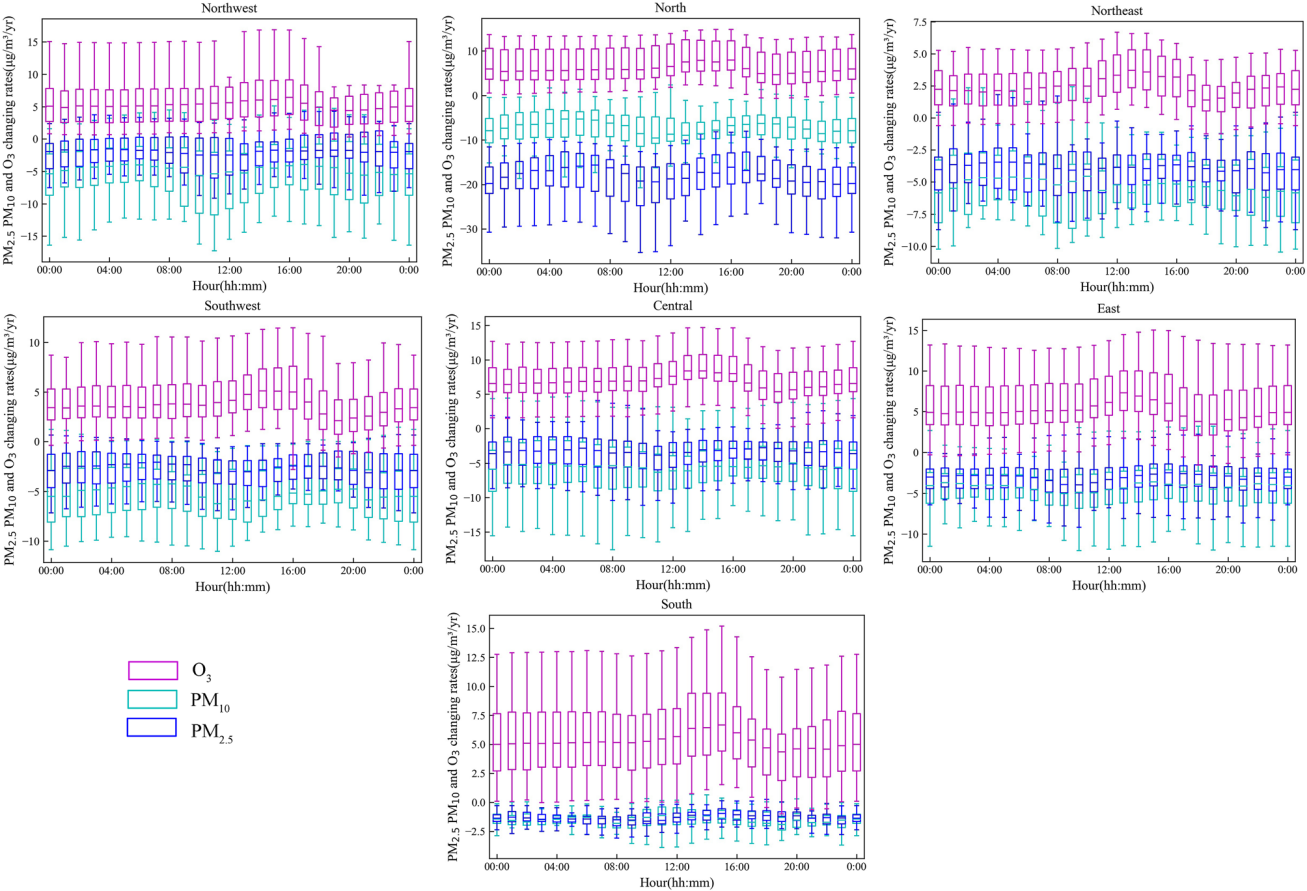
Box plots of hourly three major pollutant rates across UAs.

As seen from the above analysis, the spatial distribution of changes and the concentrations of air pollutants shared a location similarity between adjacent UAs. Furthermore, to profile the diurnal changes in pollutant concentrations during the 13th Five-Year Plan, the 24-hourly variations were calculated in seven directions. The results are shown in Fig. 6. From 2015 to 2019, the rates of O_3_ concentration in any hour significantly increased compared to particulate matter (PM) pollution, which was consistent with the average annual variation trends above. Overall, diurnal trends in O_3_ concentration at UAs in all directions also showed a single peak, with the largest increments concentrating between 13:00 and 16:00, due to more frequent heat waves in recent times^50^ and the fact that higher temperature, lower relative humidity, and stronger solar radiation at 16:00 favor more secondary pollutant production^9,51^. A similar hourly variation, which daytime O_3_ increased more significantly than that of nighttime in Beijing, has been reported^5^. However, the peak O_3_ pollution spatially varied, exhibiting a relatively earlier trend in the southeastern region than in the northwestern region because of the Earth's rotation, making the eastern area receive direct sunlight first. The distance between the whiskers of the box plot in the southern city is the largest compared with the others, indicating that ground-level ozone concentration variations presented great dissimilarity in southern cities.

For PM pollution, there was a significant downward trend in all UAs. PM_2.5_ reduction is more obvious in rush hours (10:00 and 22:00), which reveals the response to aggressive actions by the government to restrict motor vehicles^51^. In addition, we noticed that the ‘coal-to-gas and coal-to-electricity’ transformation has substantially improved air pollution, particularly in northern China. It seems clear from these figures that the changing rate of PM_2.5_ in northern regions was significantly higher than that of PM_10_.

### Spatial centroid variations of air pollutants

**Figure 7:**
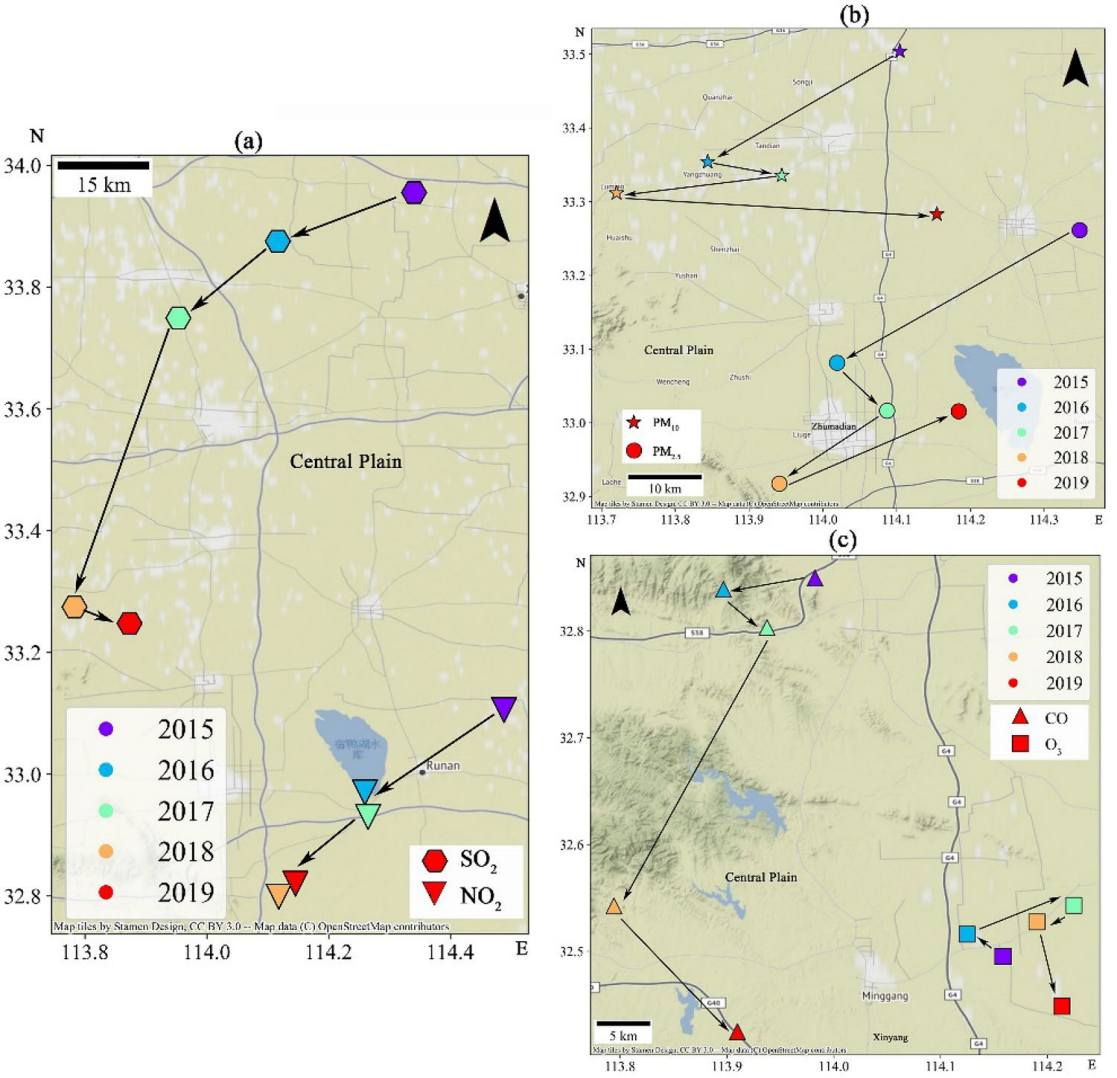
Moving path of the centroids of six air pollutants. (We marked the centroid of PM_2.5_ pollution with circle, PM_10_ with star, O_3_ with square, CO with triangle_up, NO_2_ with triangle_down and SO_2_ with hexagon.). This figure was generated with python 3.7.6 based on geopandas and contextily packages. The source of the base map from contextily package is ‘http://{s}.tile.stamen.com/terrain/{z}/{x}/{y}.jpg’.

As far as pollution centroid are concerned, their trends indicate that the ambient air pollutants are gradually moving southward and mainly concentrated in the Central Plains UA from 2015 to 2019 (Fig. 7). We noticed that the centroids of PM_2.5_ and PM_10_ showed similar movement trends, which were both firstly located in the north of the Zhumadian city in Henan Province, and following to the southwest sharply about 30 km, and then slightly shifted towards the southeast, and finally located in the south of the starting centroid. In other words, PM pollution have moved towards the south during China's 13th Five-Year Plan period, indicating that the north/northeast/northwest UAs have dropped significantly than that in the southern UAs. Consistent with the results of the Shi et al. (2019), our results show that the PM governance in China is indeed effective^52^ (Fig. 7a).

With respect to the SO_2_ and NO_2_ pollution, they were offset southwest by nearly 100 and 50 km, respectively (Fig. 7b). These two pollutants have the largest offset, which may suggest that the decline of SO_2_ and NO_2_ pollution in northeast is greater than that of other pollutants. These findings have also been evidenced by Zhang et al. (2019)^49^ and Cui et al. (2021)^53^.

With regard to the O_3_ pollution, the centroid spatial movement trend was weak and mainly located in the north of Xinyang city in Henan Province. During the 13th Five-Year Plan period, the control of ozone pollution is not obvious. Therefore, ozone pollution become the focus of the 14th Five-Year Plan exceeded PM_2.5_^54^. In term of CO pollution, it moved sharply south in 2017, indicating that CO pollution in the north has been effectively controlled since 2017 (Fig. 7c).

### Sector emission source impacts on primary pollutants

**Figure 8:**
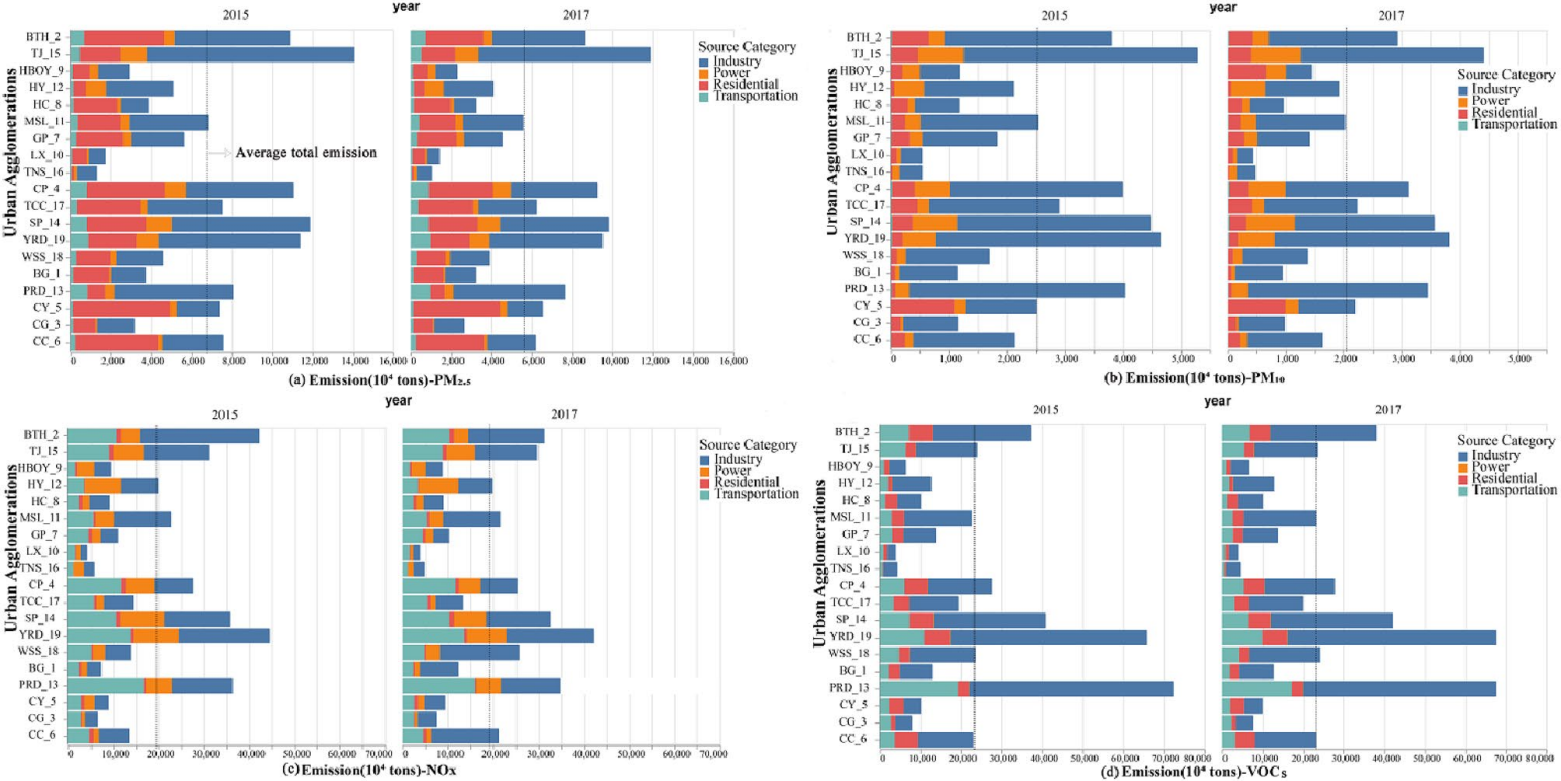
Average sector emission of pollutants in 19 UAs.

Due to the availability of data, statistics on industry emissions are limited to 2015–2017. The average annual emission value of agricultural sources was too small to provide statistical significance. Figure 8 only shows the contribution of four sector sources (industry, power, residential and transportation) to pollutants in 19 UAs from 2015 to 2017, where the dotted line represents the average of total emissions. As a result, near-surface ozone pollution is a secondary pollutant produced by the photochemical reaction of a series of precursors under the action of solar radiation. In particular, VOCs and NO_x_ are important precursors for ozone pollution^19^. Therefore, we explore the emissions of these two important precursors in different UAs.

Sector pollution sources directly cause increases in PM_2.5_, PM_10_, nitrogen oxides, volatile organic compounds and other pollutants in the atmosphere^55^. As seen from Fig. 8a, PM_2.5_ is mainly derived from industrial sources, and the main high value areas are TJ, YRD and SP UAs. Meanwhile, in the three UAs, the total contribution of anthropogenic emission sources to particulate pollution declined the fastest, which is largely related to government intervention, industrial transformation and environmental governance. Approximately 50–75% of PM_2.5_ in the northern UAs comes from industrial sources, while PM_2.5_ in the southwestern UAs mainly comes from residential sources. This result is similar to the analysis of PM_2.5_ sources in BTH in 2013 by Li et al. Regarding PM_10_, industrial sources have the greatest influence on PM_10_ in all UAs (Fig. 8b). There is no significant regional difference in the impact of traffic sources on PM_2.5_ and PM_10_. The contribution of traffic sources to PM_2.5_ in 19 UAs is 5–7% on average, and PM_10_ tends to 0. By using the receptor model method, Huang et al.^56^ found that the contribution of gasoline dust to total suspended particulate matter (TSP) in Changzhou was less than 1%, confirming that exhaust gas was not the main contribution source of PM_2.5_. In each UA, the cumulative emission of PM_2.5_ is consisted of the four average emissions caused by anthropogenic sources, which is approximately 2.5 times of that of PM_10_ (Fig. 8a,b). Here the emissions of pollutants from different sources were calculated, and the cumulative emission were all decreasing. However, we did not use the atmospheric transmission model to simulate pollutant concentration, so different from Zhang’s research^57^ on PRD, we could not get how various control measures and policies affect the monitoring concentration of different urban agglomerations.

Among the 19 UAs, NO_x_ and VOCs mainly come from industrial and traffic sources, with less contribution from residential and power sources (Fig. 8c,d). During the study period, the NO_x_ emissions of most UAs decreased, but the CY, CG and CC UAs in the southwest, WSS in the eastern and BG UAs all showed an increasing trend of NO_x_. This is mainly concentrated in the increase in industrial sources, which can also be seen from the increase in industrial production in the south. The contribution rate of power sources to VOCs in UAs is low and can be ignored.

From the perspective of time series changes, the contribution rate of anthropogenic emission sources to particulate pollution is decreasing, but for gas pollutants, nearly half of UAs show an increasing trend of VOCs and NO_X_. In addition, VOCs high value areas are mainly concentrated in the YRD, PRD, BTH and other state-level UAs. In regional joint prevention and control, different UAs have different priorities to prevent and control sector emission of pollutants.

### Limitations of this study

As mentioned in the literature reviews, air pollution is not only affected by social activities and atmospheric emissions, but also by the impacts of meteorological factors^6,44^. In previous studies, we have specially studied the impact of meteorological elements on the YRD UA and found that the winds blowing to YRD (southeasterly & northwesterly) have opposite effects on air quality. This study only visualized the emission source inventory of pollutants at urban agglomeration scale. A number of work remain to be done in future research. Meteorological factors (temperature, wind speed, wind direction and etc.^58,59^) and chemistry transport model (such as integrated source apportionment method^60–62^) were not taken into account, so the contribution of anthropogenic sources to air pollutant concentration and trans-regional transmission of pollution were not realized in this study. Thus, it is necessary to further explore the impact of the combination of social and meteorological activities on air pollution.

## Conclusions

Other studies conducted on the temporal and spatial variations in air primary pollutants in 19 UAs during the 13th Five-Year Plan are not sufficient. Based on air quality monitoring data and using mathematical statistics and cartography, this paper analyzed the spatiotemporal characteristics of primary pollutants over 19 Chinese UAs. Although we have achieved remarkable results in pollution control during the 13th Five-Year Plan period, PM and O_3_ pollution are still serious problems in China. Returning to the question posed at the beginning of this study, it is now possible to state that the main conclusions are as follows:

1. Generally, from the daily changes of all primary pollutants, 37% of the primary pollutants had no shift during the 13th Five-Year Plan period. PM_2.5_, PM_10_ and O_3_ were the main primary pollutants, especially PM_2.5_. The proportion of clean air days has increased significantly. NO_2_ pollution was rendered nonsignificant.

(2) From the perspective of monthly distribution of primary pollutants, clean days (where AQI < 50) occur mainly in South and Southwest China during summer. As the primary pollutant, PM_2.5_ is primarily concentrated in December, January and February, and PM_10_ is primarily concentrated in October–November and March–April. The O_3_ pollution in the PRD and BG is mainly concentrated from August to November. The northern, northwestern, northeastern, central and eastern UAs have similar monthly primary pollutant distributions.

(3) In terms of air quality differences in UAs, the annual average AQI showed a gradually decreasing trend outward, taking the Central Plain as the center. The Central Plain has the highest annual average AQI, and the particulate matter pollution and ozone are both high, suggesting that the control effect is not obvious in the central region. The average annual PM_2.5_ and PM_10_ concentrations decreased, most significantly in HC and MSL in the northeast, while there was little difference in annual ozone concentration. In contrast, the annual mean ozone pollution displayed the greatest enhancement, while PM_2.5_ and PM_10_ showed a minimal decrease in TJ UA in northern China. BTH UA had the largest reduction of PM_2.5_ concentration and the ambient air pollutants are gradually moving southward and mainly concentrated in the Central Plain from 2015 to 2019.

(4) There were significant differences in the intensity and direction of each sector emission source in nineteen UAs. The total amount of PM discharged from the emission sources of Taiyuan-Jinzhong, Yangtze River Delta and Shandong Peninsular was much higher than those of others. PM_2.5_ and PM_10_ are mainly derived from industrial sources. The contribution rate of anthropogenic emission sources to particulate pollution is decreasing, but for gas pollutants, nearly half of UAs show an increasing trend of VOCs and NO_X_.

Based on the above findings, this paper proposes the following policy recommendations. First, it is necessary to pay more attention to emission reduction efforts in national UAs. Second, PM and ozone pollution are important pollution types in UAs in China, and they should be coordinated in prevention and control. The government should strengthen regional joint prevention and control and establish a compensation and incentive mechanism for cities with weak economies. Third, different UAs should formulate differentiated PM and O_3_ emission reduction strategies according to their own social and economic conditions. Industrial emissions are still an important source of air pollution, and the industrial structure should be optimized based on local resources. We will encourage energy enterprises to develop energy and introduce new energy technologies. We will improve cleaner production technology and build a monitoring platform for urban greening.

## Data Availability

Data and materials are available from the authors upon request.
